# Optimizing ensemble NV^−^ spin properties of fluorescent diamond microparticles by systematic low pressure high temperature annealing

**DOI:** 10.3389/frqst.2025.1709220

**Published:** 2025-11-26

**Authors:** Nicholas Nunn, Antonin Marek, Marco D. Torelli, Alex I. Smirnov, Olga A. Shenderova

**Affiliations:** 1 Adámas Nanotechnologies, Inc., Raleigh, NC, United States; 2 Department of Chemistry, North Carolina State University, Raleigh, NC, United States

**Keywords:** photoluminescence, optically detected magnetic resonance (ODMR), electron paramagnetic resonance (EPR), NV centers, high temperature annealing, quantum sensing, spin relaxation, diamond particles

## Abstract

Low pressure high temperature annealing is a means for driving nitrogen and defect diffusion in diamond to reduce internal lattice damage without the need for technically complicated high-pressure cells. Herein, we perform a systematic time (5, 15, and 30 min) and temperature (1200 °C–1800 °C) study of effects of low-pressure high temperature annealing on photoluminescence, spin concentrations, and spin relaxation properties of NV centers in *ca*. 3 μm synthetic type 1b diamond particles. Annealing in the temperature range of *ca*. 1400 °C–1700 °C for even 5 min leads to a higher optically detected magnetic resonance contrast as compared to standard annealing at 900 °C for 2 h. Particles annealed at 1700 °C for 5 min exhibit a contrast close to about 13% as compared to about 9% for those annealed at 900 °C for 2 h. A reduction in the zero-field splitting strain parameter from *E* ≈ 4.5 MHz to ≈2.5 MHz and spectral linewidth from Δ*ν* ≈ 7 MHz to ≈4 MHz are observed even after 5 min annealing at 1700 °C. Improvements in these spectral parameters resulted in a roughly 2-fold reduction in the noise level of temperature monitoring experiment utilizing an ensemble of NV centers in the particles. Annealing in the temperature range of 1600 °C for 15 or 30 min or 1700 °C for 5 min resulted in NV *T*_1_ relaxation times approaching *ca*. 5 ms typically observed for bulk diamond. Quantitative electron paramagnetic resonance (EPR) allowed for estimations of thermal activation energies of paramagnetic center annihilation. Monitoring the primary defect concentration (P1 and other defects with half integer spins) and utilizing second order kinetic modeling, an activation energy of 3.63 ± 0.28 eV was estimated. Alternatively, using the NV half field EPR signal and first order kinetic modeling, a similar activation energy 3.89 ± 0.29 eV was estimated.

## Introduction

The nitrogen-vacancy (NV^−^) center in diamond is a fluorescence based platform for quantum sensing and quantum information science (Rembold et al., 2020; Aslam et al., 2023) due to the high levels of optically induced electronic spin polarization and long spin coherence times that persist even at room temperature. The NV^−^ center has a ground electronic triplet spin state (*S* = 1) with zero-field splitting (ZFS) of *D* ≈ 2.87 GHz. In the absence of an external magnetic field the spin states *m*_S_ = ±1 are degenerate and the allowed transitions between the *m*_S_ = 0 and *m*_S_ = ±1 levels occur at a resonance frequency of ≈2.87 GHz. These transitions can be readily observed by conventional electron paramagnetic resonance (EPR) with inductive detection or optically as a drop in the fluorescence emission intensity when the sample is irradiated with microwave at the resonance frequency. Changes in the diamond lattice, such as those induced by pressure or temperature affect the electronic structure of NV^−^ center and interactions between the spins responsible for ZFS, causing shifts in the resonance frequency. Degeneracy of the *m*_S_ = ±1 states is lifted by static magnetic fields causing the resonance lines to split while alternating magnetic fields shorten the lifetime of the spin states. These changes in magnetic resonance parameters can be measured with high accuracy by optically detected magnetic resonance (ODMR), making NV^−^ centers a promising platform for quantum sensing of pressure, temperature, and stochastic magnetic fields such as those induced by nearby nuclear or electronic spins. The latter external spins can be manipulated by external radiofrequency and/or microwave pulses, and such an approach was exploited for optically detected nanoscale nuclear magnetic resonance (NMR) (Bruckmaier et al., 2023; Bucher et al., 2020; Holzgrafe et al., 2020) or EPR (Qin et al., 2023; Hall et al., 2016).

As applications of NV^−^ centers in quantum sensing continue to grow, so are the needs for fabrication of diamond quantum materials with spin properties of NV^−^ centers tailored to specific applications. A common fabrication scheme of NV^−^ centers in synthetic diamond consists of two steps: (1) irradiation with a high energy electron beam (e-beam) to create vacancies in the diamond lattice and, (2) subsequent annealing at *ca*. 800 °C or above to promote diffusion of the vacancies which combine with substitutional nitrogen atoms and form stable NV^−^ centers. While the existence of many different ‘color centers’ in diamond has been documented, those arising from nitrogen in its substitutional form are predominant in synthetic type 1b diamond (Breeding and Shigley, 2009), which is widely used for producing fluorescent diamond particles (FDPs). In a neutral charge state (N_s_^0^), substitutional nitrogen centers are paramagnetic (*S* = ½) and are readily identified by their characteristic EPR spectrum. These defects known as P1 centers are typically found at 1–2 orders of magnitude higher concentration than NV^−^ centers and, therefore, can diminish NV^−^ quantum properties, such as spin coherence times, via dipolar interactions (Bauch et al., 2020). High nitrogen concentrations also degrade NV^−^ fluorescence emission intensity due to enhanced non-radiative tunneling pathways for electronic relaxation (Capelli et al., 2022; Shames et al., 2017a).

Spin properties of NV^−^ centers in diamond particles can also be affected by the processing methods. For example, additional paramagnetic defects (vacancies) can be created by electron irradiation, and such defects may not anneal out completely at typically utilized temperatures. Lattice deformation from crushing of larger diamond particles can also change electronic configuration of NV^−^ centers that manifests in an increase in the rhombic (transverse) anisotropy of ZFS described by the parameter *E*. A distribution of lattice deformations is also expected to lead to a distribution of the ZFS parameters *D* and *E* causing an additional broadening of EPR and ODMR resonance lines. This is undesirable for quantum sensing applications such as temperature measurements, for which the width of the ODMR resonance lines must be as narrow as possible for higher sensitivity. An increase in magnetic anisotropy also provides an additional relaxation pathway for the electronic spin system. For quantum sensing applications, it is desirable to have NV^−^ centers with long spin-lattice (*T*_1_), spin coherence (*T*_2_), and inhomogeneous dephasing/spin memory (*T*_2_*) times. In addition, particles with sufficient brightness of NV^−^ ensembles (*i.e*., photon count rates) are required to enable optical detection.

Some of the primary pathways to improving NV^−^ spin properties are by reducing concentration of paramagnetic defect centers and the lattice strain. Control of the paramagnetic center concentration can be achieved by various means, including but not limited to: (*i*) adjustment of the nitrogen content during synthesis, (*ii*) optimization of the irradiation fluence and energy, and (*iii*) optimization of the annealing temperature and time. Previously, investigators focused on decreasing and optimizing nitrogen content in chemical vapor deposition (CVD) (Healey et al., 2020; Wood et al., 2022; Luo et al., 2022) and high pressure high temperature (HPHT) (Healey et al., 2020) grown diamond plates and particles (Nunn et al., 2025). Several studies of effects of e-beam fluence and energy can be also found in literature (Luo et al., 2022; Nunn et al., 2025; Shames et al., 2017b). While some studies investigated the role of annealing temperature and time on the NV spin properties, these studies were typically limited to maximum temperatures of about 1200 °C and often were focused on bulk diamond plates but not particulate diamond. For example, Naydenov et al. (2010) reported that the fraction of NV centers in ion implanted CVD diamond plates exhibiting coherence times (*T*_2_) 50 μs or longer was increased from ~40% to >80% following annealing under nitrogen at 1200 °C for 12 h. The increase in coherence times was attributed to removal of the implantation induced defects (*e.g*., O1, R4, R5, R9, and R10 defects). Yamamoto et al. (2013) reported on high temperature annealing (1000 °C for 2 h) of 10 MeV ^15^N ion implanted ^12^C enriched single crystal CVD diamond and achieved room temperature spin coherence (*T*_2_) times of 2 ms. Tetienne et al. (2018) also reported the impacts of annealing in spin ensembles of near surface NV centers in ion implanted CVD diamond plates. Following annealing at 1200 °C, the authors of that study used optically driven double electron-electron resonance (DEER) and cross relaxation spectroscopy (*T*_1_-EPR) and reported an increase in *T*_2_ coherence times and Rabi contrast, as well as a reduced ODMR linewidth. This was ascribed to a removal of (*i*) fast relaxing paramagnetic species such as the R4/W6 center (neutral divacancy, V_2_^0^) formed upon ion implantation, and, possibly, (*ii*) multivacancy chain defects such as the R5 center (neutral trivacancy, V_3_^0^). Regarding studies involving diamond particles, Dei Cas et al. (2019) demonstrated the potential of low pressure high temperature (LPHT) rapid thermal annealing (RTA) of e-beam irradiated 20 μm particles in the temperature range from *ca*. 500 °C–2000 °C, including annealing of 140 nm particles at 1900 °C for 1 min. Most of the protocols were limited to annealing for 10 min or less although a single 50 min annealing at 1700 °C was performed. The primary focus of the study was on the luminescence of the annealed diamond particles with less attention paid to spin properties. However, EPR analysis showed a reduction in the total concentration of *S* = 1/2 defects from 412 ppm after irradiation to 32 ppm after 1 min annealing at 1900 °C and an estimated order of magnitude elongation in the spin lattice relaxation time. Annealing at 1900 °C also reduced the NV^−^ center concentration to <1 ppm (Dei Cas et al., 2019). A subsequent study reported on LPHT annealing of e-beam irradiated 40 μm particles (Shenderova et al., 2020). The electron fluence and energy (3 × 10^18^ e/cm^2^, 1 MeV) were lower as compared with the initial study of 20 μm particles by Dei Cas et al. (2019) (1 × 10^19^ e/cm^2^, 5 × 10^19^ e/cm^2^, 3 MeV). Milder irradiation conditions provided a greater luminescence homogeneity among the particles, and, interestingly, a lesser impact of high temperature annealing on the estimated spin lattice relaxation times was observed from analysis continuous wave (CW) EPR microwave power saturation curves. This was attributed to a lower diamond lattice damage due to milder irradiation conditions (Shenderova et al., 2020). A third RTA study showed that magnetic field induced fluorescence contrast of 20 μm particles irradiated to a fluence of 1.5 × 10^19^ e/cm^2^ and annealed at 1740 °C for 8 min increased about fourfold, *i.e*., from ~5% to ~20% (Torelli et al., 2020). Furthermore, 20 μm particles annealed at 1720 °C for 20 min exhibited a 36-fold enhancement of optically driven ^13^C hyperpolarization compared to the same particles annealed at 850 °C for 2 h (Gierth et al., 2020). While these studies do indicate that higher temperature annealing can enhance NV spin properties, to the best of the authors knowledge, no systematic study of RTA parameters combined with ODMR characterization has been reported so far. The focus on the ODMR characterization is practically important because ODMR is the main platform for many quantum sensing applications of NV^−^ centers.

To fill this gap in the current literature, we therefore conducted a systematic evaluation of the impact of annealing parameters in the high temperature regime by investigating the annealing of 3 μm diamond particles across a range of temperatures (1200 °C–1800°) for 5, 15, and 30 min. The main objective was to determine whether optimization of temperature and time of RTA treatment would improve NV^−^ spin properties without sacrificing much the NV^−^ concentration. The latter was of a particular concern because annealing at higher temperatures for longer duration (*ca*. 1600 °C for 2 h) was shown to result in a significant loss in NV^−^ concentration from about 3–4 ppm to <0.5 ppm resulting in a lower fluorescence signal (Nunn et al., 2023). The particles were also characterized by EPR to estimate activation energies of paramagnetic center annihilation in the lattice.

## Experimental methods

**FIGURE 1:**
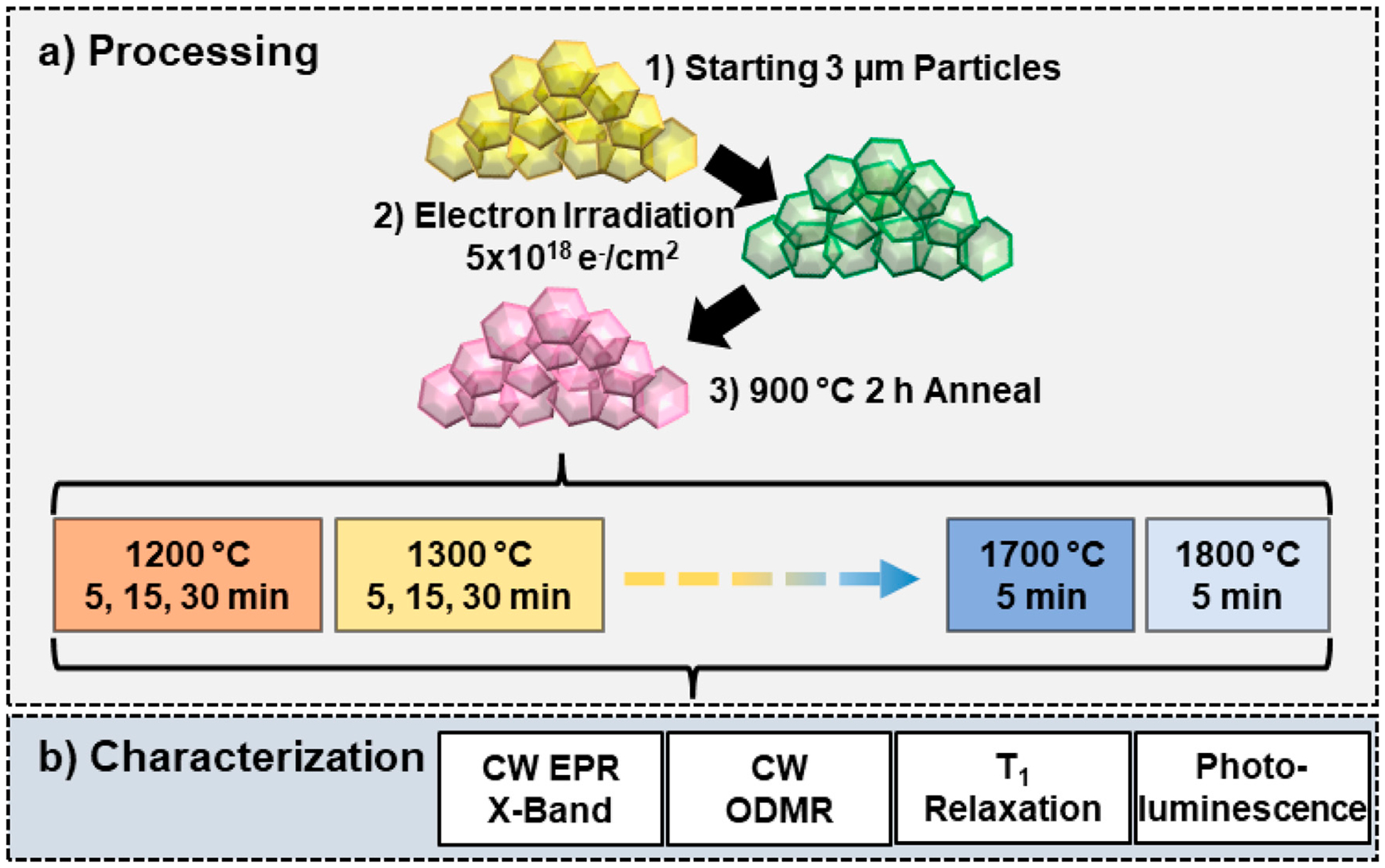
Experimental design for 3 μm diamond particle annealing study. (a) The starting 3 μm particles were irradiated with 3 MeV electrons to a fluence of 5 × 10^18^ e^−^/cm^2^ and subsequently annealed at 900 °C for 2 h *in vacuo*. The annealed particles were then subjected to further high temperature annealing from 1200°C–1800 °C in 100 °C increments for fixed times (5, 15, or 30 min). Duration of annealing at 1700 °C and 1800 °C was limited to 5 min to avoid further graphitization at these temperatures. (b) Each sample was characterized with CW EPR, CW ODMR, optical *T*_1_ relaxation, and photoluminescence (PL) emission to identify defects present, determine spin concentrations, and evaluate optical properties of the samples as a function of annealing temperature and time.

Figure 1 illustrates experimental design for this study. Particle sizes of *ca*. 3 μm were selected because they were anticipated to be less susceptible to graphitization at high temperature than small particles–an important consideration for both producing sufficient quantity of annealed particles and reducing cost. Moreover, larger particles can be further milled to smaller sizes.

### Materials

Type 1b synthetic diamond particles *ca*. 3 μm in diameter (Adámas Nanotechnologies, Raleigh, NC, United States) were irradiated with 3 MeV electrons to a fluence of 5 × 10^18^ e^−^/cm^2^ and annealed in a vacuum tube furnace (CTF 17/600, Carbolite Gero Ltd., Hope Valley, United Kingdom) at ≈10^−6^ Torr at 900 °C for 2 h to form NV^−^ centers and then oxidized in air at 550 °C for 2 h to remove surface graphite. 150 mg portions of these particles were then subjected to high temperature annealing in the range of 1200 °C–1800 °C for 5, 15, or 30 min in an all-graphite low pressure high temperature (LPHT) rapid thermal annealing furnace (High T Technologies, New York, NY, United States). For the 1700 °C and 1800 °C treatments, only 5-min annealing was performed due to significant graphitization and mass loss of the diamond particles observed at these temperatures (Table 1). A photograph of diamond powders following 5 min high temperature annealing in the temperature range of 1500 °C–1800 °C illustrates a progressive graphitization with temperature (Supplementary Figure S1, Supplemental Information). After the annealing, the particles were oxidized with molten potassium nitrate (KNO_3_, ACS Reagent Grade ≥99%, Sigma Aldrich, St. Louis, MO, United States) in air at 550 °C, similar to previously reported literature (Havlik et al., 2013), to remove graphitic phase carbon.

### Optically detected magnetic resonance

CW ODMR spectra were collected using an in-house built system configured with an Olympus IX71 epifluorescence microscope (Olympus Corporation of the Americas, Center Valley, PA, United States). Samples were prepared by drop casting aqueous suspensions of the particles (*ca*. 5 μL, 10 mg/mL) onto a glass coverslip. Once dried, the particles ensemble CW ODMR spectra were collected under continuous optical illumination and microwave driving. Light excitation was provided from a pE-300 white LED (CoolLED, Ltd., Andover, United Kingdom) filtered through a 562/40–25 nm bandpass excitation filter (Semrock FF01–562/40–25, IDEX Health and Science, LLC, West Henrietta, NY, United States). Emission light was passed through a beamsplitter (Semrock FF593-DI03–25 × 36) and 650 nm long-pass filter (FELH0650, Thorlabs Inc., Newton, NJ, United States) to a fiber coupled avalanche photodiode (APD) (APD440A, ThorLabs Inc.). Electrical output from the APD was captured on an oscilloscope (MDO4104C, Tektronix, Beaverton, OR, United States) and downloaded to a PC. A microwave signal (*ca*. 2.87 GHz) from a signal generator (SRS SG384, Stanford Research Systems, Inc., Sunnyvale, CA, United States) was amplified to ≈2 W by an amplifier (ZHL-2W-63-S+, MiniCircuits, Brooklyn, NY, United States) and delivered to the sample via a Thincol EMI near field RF ‘sniffer’ probe (Amazon, Seattle, WA, United States) in a ring configuration affixed to the stage of the microscope. The highest levels of photoluminescence magnetic field contrast were measured under low LED intensity (*ca.*, 10% setpoint on LED controller) and a higher microwave power (−5 dBm or 0.3 mW). Therefore, all CW ODMR spectra were collected at the above conditions for a relative comparison. The LED power was estimated using a PM400 Optical Power Meter and an S401C Thermal Power Sensor Head (both from ThorLabs Inc.). Results of these power measurements are shown in Supplementary Figure S2 of Supplemental Information. A power density of ≈16 mW/mm^2^ was estimated for the 10% LED setpoint and 40× magnification was used for these measurements. ODMR spectra were fitted to a superposition of two Lorentzian functions to determine full width at half maximum (FWHM) of the signals and the strain parameter *E*.

### *T*_1_ relaxation measurements

For *T*_1_ relaxation measurements, light excitation pulses were generated by the pE-300 white LED triggered by an arbitrary waveform generator (AWG) (SDG1025, Siglent Technologies NA, Inc., Solon, OH, United States). The optical excitation and emission filters for *T*_1_ measurements were kept the same as in CW ODMR experiments. In a typical measurement, the NV^−^ spins were initialized with a several ms pulse which length was optimized to ensure full polarization. The optimization consisted of monitoring the polarization buildup trace (the signal output of the APD) as a function of the initialization pulse length. The pulse duration at which no further buildup of the polarization was observed is then chosen for the initialization pulse. This initialization pulse was followed by a progressively incremented delay time *t*. The signal collected during the first ~100 μs of the next initialization pulse was linearly extrapolated to determine the signal decay after the preceding pulse. Equation 1 was used to quantify the fluorescence intensity I as an output voltage of the APD as a function of the delay time *t* and fitted to a stretched exponential function containing a baseline term:

(1) $$I(t)=I_{\text{polarized}}\;e^{{\left(-\frac{t}{T_{1}}\right)}^{p}}+Bt+I_{\text{relaxed}}$$

where *I*_polarized_ is the maximum increase of fluorescence due to light polarization, *B* is a parameter to account for a slow linear baseline drift, and *I*_relaxed_ represents the fluorescence signal in the fully relaxed state (*i.e*., *t* → +∞).

We also define a *T*_1_ contrast parameter $\left(C_{T_{1}}\right)$ as:

(2) $$C_{T_{1}}=\frac{I_{\text{polarized}}-I_{\text{relaxed}}}{I_{\text{polarized}}}$$

This contrast is used as a metric to compare the amounts of optically induced NV polarization for different samples.

### Continuous wave electron paramagnetic resonance

Continuous Wave EPR spectra were measured at X-band (9.87 GHz) using a Bruker Elexys-II E500 spectrometer (Bruker Biospin, Billerica, MA United States) equipped with a Super High Sensitivity Probehead installed at North Carolina State University (Raleigh, NC, United States). Spin concentrations were estimated by comparing double integrals of *g* ≈ 2.0 EPR signals with that of a reference sample of 1 μm diamond particles containing an estimated 3.5 × 10^18^ spins/g of primary defects and 9.7 × 10^16^ spin/g of NV^−^ (Adámas Nanotechnologies, Raleigh, NC, United States). Spline interpolated baseline corrections of EPR spectra were carried out using XEpr software (Bruker) or MATLAB^®^ (The Math Works, Inc., Natick, MA United States) using the baseline fit function (Mathworks, 2024) as needed. NV^−^ spin concentrations were estimated from double integration of the best fits of the experimental *g* ≈ 4.2 (*i.e*., half-field) spectra using EasySpin software (Stoll and Schweiger, 2006) instead of direct double integration due to a low signal-to-noise ratio of these EPR spectra corresponding to forbidden transitions.

### Estimation of thermal activation energies of defect annihilation from EPR spectra

Activation energies of annihilation of EPR-active defects were estimated from sample mass-normalized EPR double integrated intensities measured for samples after the annealing treatments for a set of temperatures and the annealing periods. We assume that temperature ramping time is short relative to the duration of the annealing and that defect annihilation occurs only during the annealing period, hence, the duration of the annealing, *t*, is the reaction time.

Both first and second order annihilation mechanisms were investigated. For the first order mechanism, the rate of annihilation of species X is given by Equation 3 and the corresponding integrated rate law by Equation 4, where [X_0_] is the initial concentration, and *k*_1_ is the first order rate constant in units of (s^−1^):

(3) $$\frac{d[\text{X}]}{dt}=-k_{1}[\text{X}]$$

(4) $$\text{ln}[\text{X}]=-k_{1}t+\text{ln}\left[{\text{X}}_{0}\right]$$

Thus, a plot of ln[X] versus the annealing time *t* should yield a straight line if the assumption of first order kinetics is valid. Second order kinetics have a corresponding rate law and integrated rate law given by Equations 5 and 6, where *k*_2_ is the first order rate constant in units of (ppm^−1^s^−1^):

(5) $$\frac{d[\text{X}]}{dt}=-k_{2}[\text{X}{]}^{2}$$

(6) $$\frac{1}{\left[\text{X}\right]}=k_{2}t+\frac{1}{\left[X_{0}\right]}$$

Once rate constants *k*_*i*_ are determined for a set of the annealing temperatures, the activation energies *E*_*a*_ of defects annihilation can be estimated from the Arrhenius relation, Equations 7, 8:

(7) $$k_{i}(T)=Ae^{-\frac{E_{a}}{RT}}$$

(8) $$\text{ln}\left(k_{i}\right)=-\frac{E_{a}}{RT}+\text{ln}(A)$$

Where *E*_*a*_ is the activation energy (J × mol^−1^), *R* is the ideal gas constant (8.314 J × K^−1^ mol_−1_), and *A* is an empirical pre-exponential factor.

### Photoluminescence (PL) characterization

Photoluminescence (PL) spectra of particles were collected under continuous blue illumination (470/20 nm excitation filter) provided from the same pE-300 white LED. This excitation wavelength was selected because it allows for simultaneous monitoring of NV and NVN related emissions. Specifically, the ratio of the PL intensities at 520 nm and 638 nm, corresponding to the NVN and NV^−^ center emissions, respectively, was monitored as a function of the annealing temperature and time.

### NV thermometry noise level measurement

Ambient temperature was monitored over the course of 1 h using either the 900 °C–2 h starting particles or the 1700 °C–5 min annealed particles. Measurements were recorded under high LED light intensity (30% setpoint) and low microwave power (−15 dBm). A previously published frequency jump modulation scheme (Singam et al., 2020) was used with our in-house instrument configuration to record the temperature. To estimate the noise level of the measurement, the raw temperature signals (Supplementary Figure S4, Supplementary Information) were corrected with a baseline spline to remove slow temperature drifts from the recorded data. Then, the noise level of the measurement was estimated by calculating standard deviations (*σ*) of the residual temperature fluctuations for each sample.

## Results and discussion

### Continuous wave EPR at X-band

**FIGURE 2:**
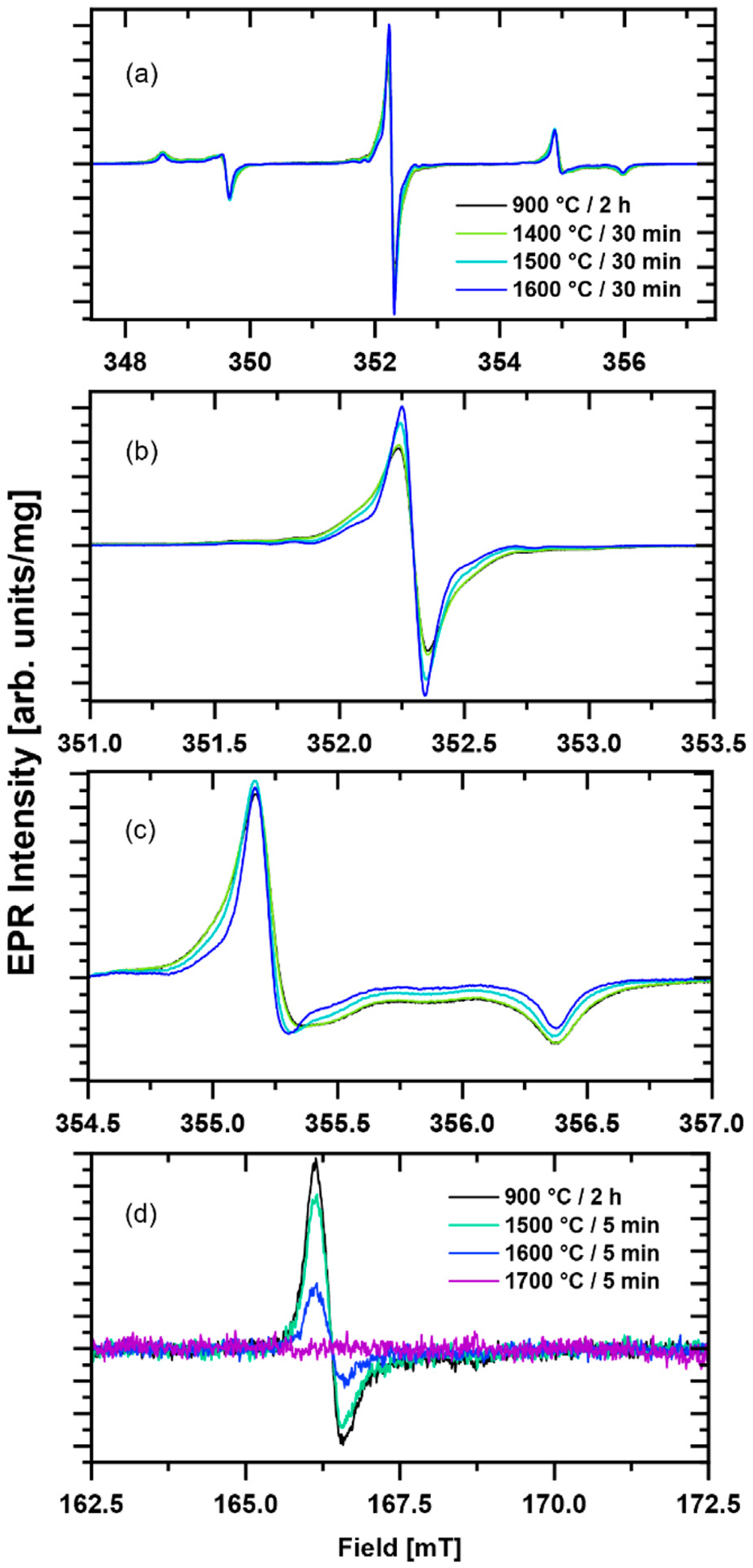
Mass-normalized experimental X-band CW EPR spectra of 3 μm diamond particles following 30 min annealing at specified temperature are compared to that of the initial particles annealed at 900 °C for 2 h. The spectrum in the *g* ≈ 2.00 region is shown as **(a)**, **(b)** is a zoom-in of the central *m*_*I*_ = 0 nitrogen hyperfine component of the P1 spectrum, and **(c)** is the region of the P1 high field *m*_*I*_ = −1 hyper fine component. Half-field EPR spectra of 3 μm diamond particles before and after 5 min annealing at different temperatures **(d)**. All EPR spectra were recorded at room temperature.

**FIGURE 3:**
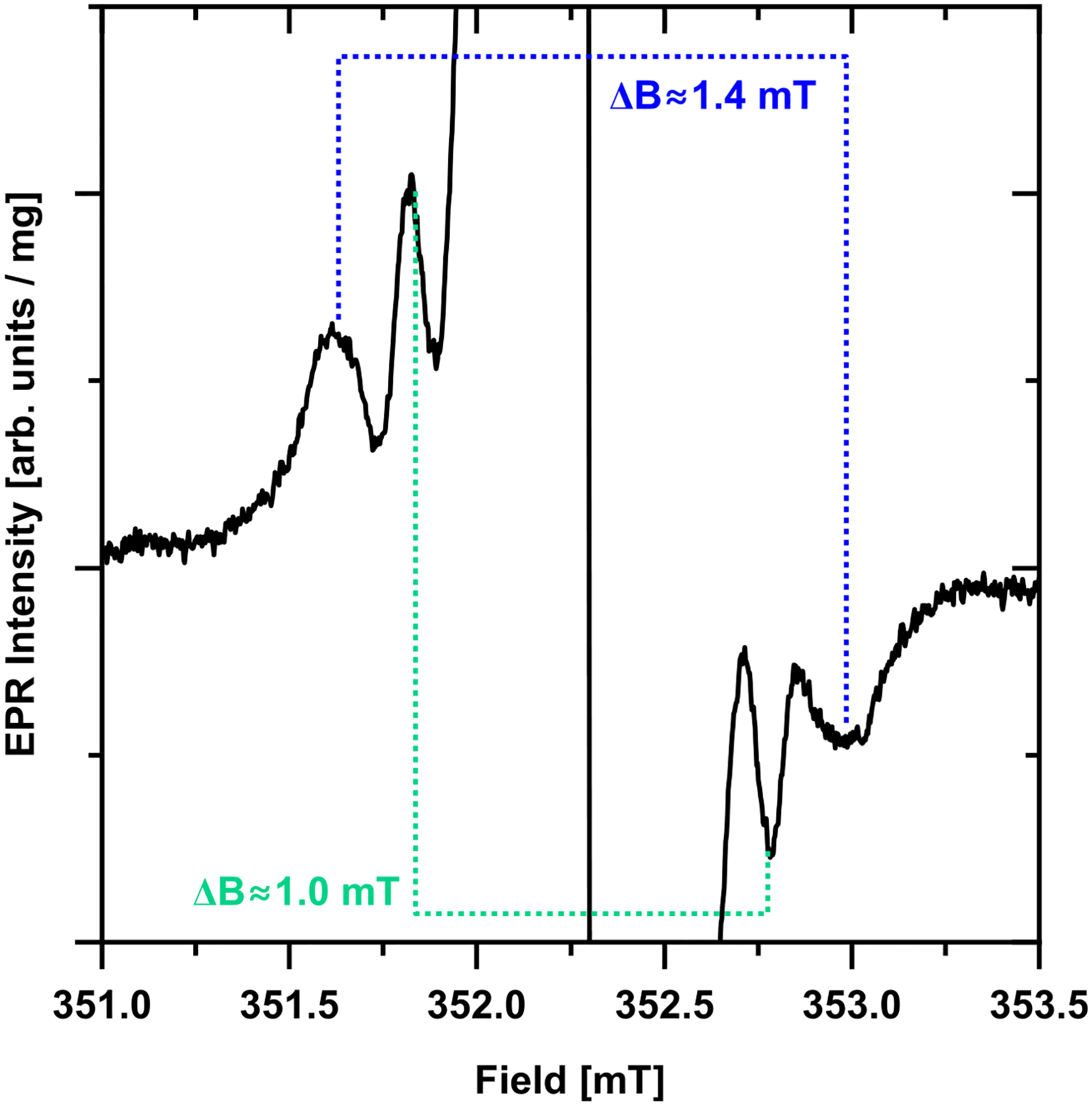
Zoom in on the *g* ≈ 2.00 region of the primary defect EPR signal of the 1600 °C/30 min annealed sample (black trace). Two pairs of weaker lines with separations of ≈1.0 mT and ≈1.4 mT are observed. These lines are likely hyperfine splitting on ^13^C present in the diamond lattice in natural abundance (1.1%) which become resolvable due to narrowing of the primary defect lines after high temperature annealing.

There are two regions of interest in the EPR spectra of the diamond particles: (1) a region at *g* ≈ 2.00 and (2) a half-field ‘forbidden transition’ region at *g* ≈ 4.20–4.30. The strongest signal in the primary defect *g* ≈ 2.00 region for the type 1b diamond used in this study originates from P1 center corresponding to substitutional nitrogen atoms although other half-integer spins such as dangling bonds (*S* = 1/2) and vacancies (*S* = 3/2) (Shames et al., 2002; Shames et al., 2015; Shames et al., 2023) are also expected to contribute. The latter ‘non-P1’ EPR lines overlap with the central nitrogen hyperfine component of the P1 signal. P1 centers and the other paramagnetic defects in this *g* ≈ 2.00 region are referred to as the *primary defects*. Representative room temperature EPR spectra of the diamond particles before and after 30 min high temperature annealing treatments at 1400 °C, 1500 °C, and 1600 °C in the primary defect region are shown in Figures 2a–c. The most notable observation is a narrowing of the EPR lines after annealing at 1500 °C and higher. This is attributed to the removal of some of the aforementioned “non-P1” related centers and a reduction in P1 center concentration as indicated by a sharpening of the outer lines corresponding to P1 *m*_*I*_ = ± 1 nitrogen hyper fine component (Figure 2c). Additional weaker lines off the central *m*_*I*_ = 0 nitrogen hyperfine component of the P1 spectrum become visible after annealing in the temperature range of 1500 °C–1700 °C. Two nearly symmetric lines with a separation of ≈ 1.0 mT (≈28 MHz) and another set of two lines with ≈1.4 mT (≈39 MHz) (Figure 3). These are likely attributable to hyperfine interaction with ^13^C present in natural abundance (≈1.1%) (Barklie and Guven, 1981). Aside from these observations, there were no other significant changes in the EPR spectra in this primary defect region for the particles after a high temperature annealing. Substantial loss of mass for the 1800 °C annealed sample made its EPR characterization impossible.

**FIGURE 4:**
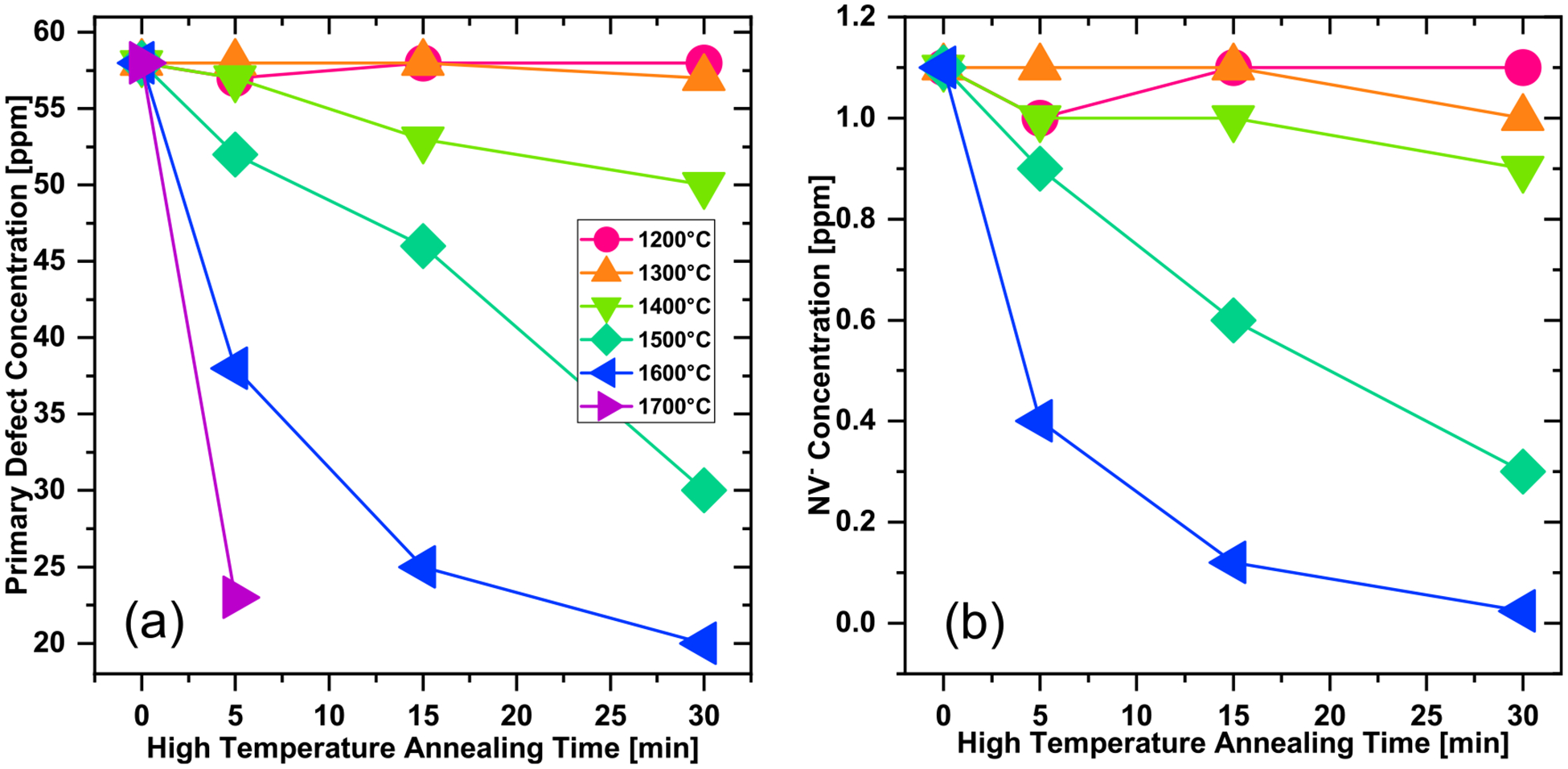
Concentration of **(a)** primary defects and **(b)** NV^−^ centers derived from quantitative EPR measurements as a function of the annealing time for annealing temperatures from 1200 °C to 1700 °C. Color coding of the temperatures is shown as an insert in **(a)**. Initial defect concentrations (0 minutes) are for the particles after e-beam irradiation and annealing at 900 °C for 2 h. Annealing at 1700 °C resulted in a significant loss of the particles yielding quantities insufficient for EPR detection of NV^−^.

Estimated P1, primary, and NV defect contents (in ppm) from double integration of spectra are summarized in Table 1. Estimated primary and NV defect concentrations are additionally plotted as a function of the annealing time for different temperatures in Figures 4a,b, correspondingly. A reduction in P1 concentration from ≈50 to ≈20 ppm is observed as the annealing temperature and duration are increased. It should be noted that the estimated P1 content in the starting particles was lower than for typical type 1b diamond, but this is because the particles were previously irradiated and initially annealed at a lower (*i.e*., 900 °C) temperature (Shames et al., 2017b; Nunn et al., 2022). The fraction of P1 in the total paramagnetic defects observed by EPR increased from about 87% in the initial particles (900 °C/2 h) to about 94% after 1700 °C–5 min annealing. Because of the dominance of P1 centers among all the paramagnetic defects, the primary effect of the high temperature annealing of these 3 μm particles is mostly likely related to a dissolution of P1 centers at *ca*. 1400 °C–1500 °C and higher temperatures. Annealing of type 1b bulk diamond in this temperature range can result in an aggregation of substitutional nitrogen to form Nitrogen-Nitrogen (N-N) pairs (the so-called, A-centers) (Collins, 1980; Jones et al., 2015; Collins et al., 2005; Collins, 1979). The presence of vacancies (*e.g*., those formed upon irradiation) could accelerate nitrogen aggregation (Collins, 1980).

A second magnetic field region of interest is *g* ≈ 4, where *forbidden* (Δ*m*_s_ = 2) *half-field* transitions of the NV^−^ center are detected (Segawa and Shames, 2020). Some representative EPR spectra are shown in Figure 2d. A substantial reduction in the half-field signal intensity is observed with increasing annealing temperature. The signal becomes comparable with noise after annealing for 30 min at 1600 °C (not shown) and is undetectable for the sample annealed for 5 min at 1700 °C (Figure 2d, magenta EPR spectrum was recorded by averaging signal for 10 min at room temperature with 8.9 mg of sample.

**FIGURE 5:**
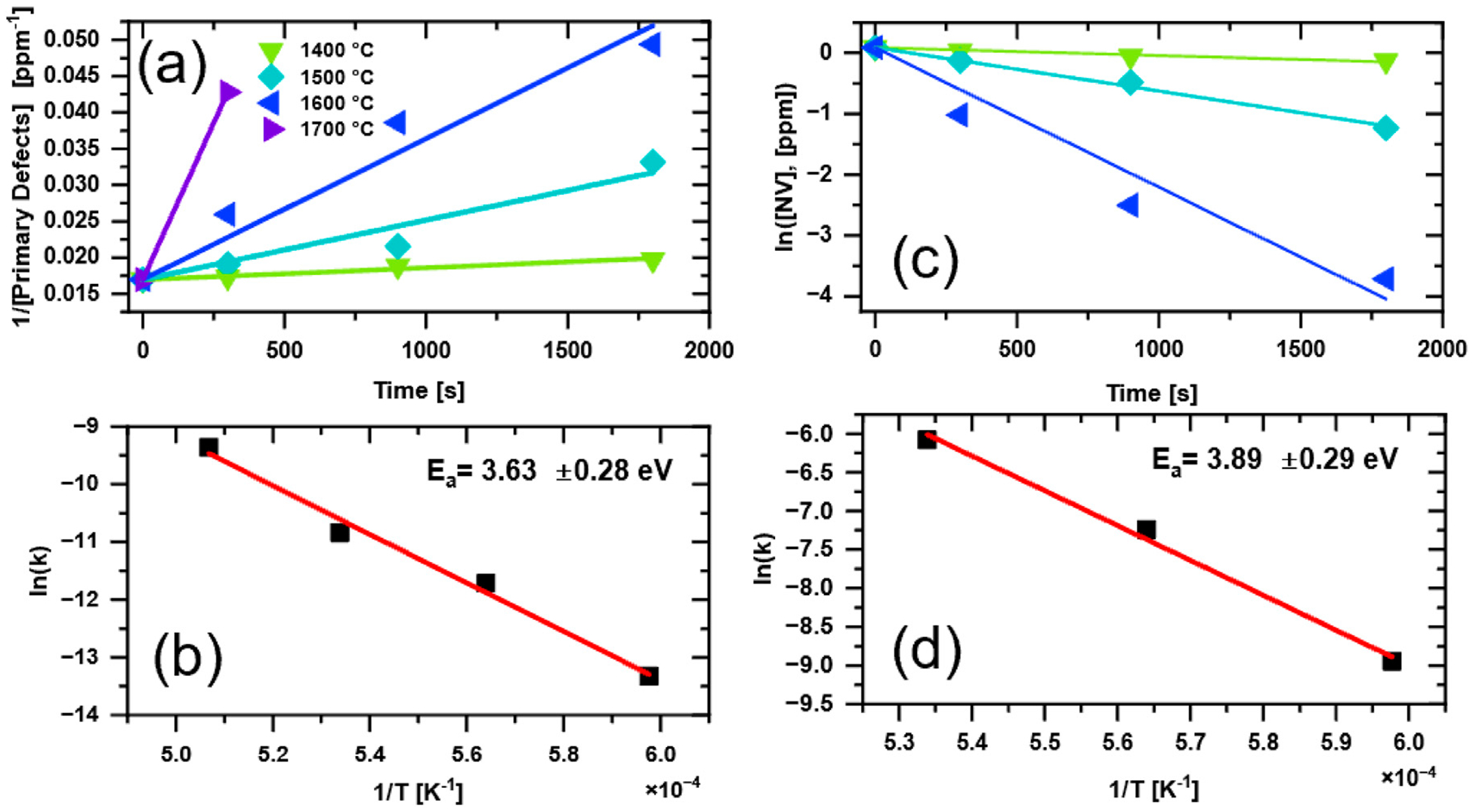
Analysis of the annealing kinetics and the corresponding Arrhenius plots for primary defect **(a,b)** and NV^−^ centers **(c,d)**. For the primary defects, second order kinetics are assumed, while first order kinetics are assumed for the NV centers. Spin concentrations were estimated from CW EPR (See Table 1). Note that the 1200 °C and 1300 °C temperatures were excluded, since the EPR results indicated no substantiative changes in the spin concentrations at those temperatures. Symbols of the same shape and color were used for **(a)** and **(c)** and the annealing temperatures are shown as an insert in **(a)**. See text for details.

The primary defects EPR signal is dominated by P1 centers for which the main mechanism of annihilation upon high temperature annealing is migration and aggregation of nitrogen defects to form N-N pairs that bear no unpaired electron and are EPR silent. Formation of N-N pairs is expected to follow second order kinetics given by Equations 5 and 6 (Chrenko et al., 1977; Evans and Qi, 1982). The second order rate constants can be estimated from the plots shown in Figure 5a and the corresponding Arrhenius plot is presented in Figure 5b yielding an estimated activation energy of *E*_a_(P1) = 3.63 ± 0.28 eV.

Analysis of changes in NV^−^ concentration upon high temperature annealing showed that a first order kinetic model fits the limited set of data well (Figure 5c). The corresponding Arrhenius plot in Figure 5d yields an estimated activation energy *E*_a_(NV^−^) = 3.89 ± 0.29 eV. These estimated values lie between that of vacancy migration energy of 2.6 eV (Davies et al., 1992) and vacancy assisted nitrogen migration of 4.5 eV (Mainwood, 1994). The close values of *E*_a_(P1) and *E*_a_(NV^−^) may indicate that the reaction rate are dominated by the same temperature activated diffusion process in the diamond lattice, such as vacancy assisted nitrogen migration. Speculation on the mechanisms at play are beyond the scope of the current data and discussion; however, these results provide some insights into defect formation/annihilation kinetics in particulate diamond. One challenge with utilizing X-band EPR for studying diamond particles is low spectral resolution in the primary defects in the *g* ≈ 2.00 region where signals from different defects overlap and are also inhomogeneously broadened by orientational averaging in the magnetic field.

### Photoluminescence (PL)

**FIGURE 6:**
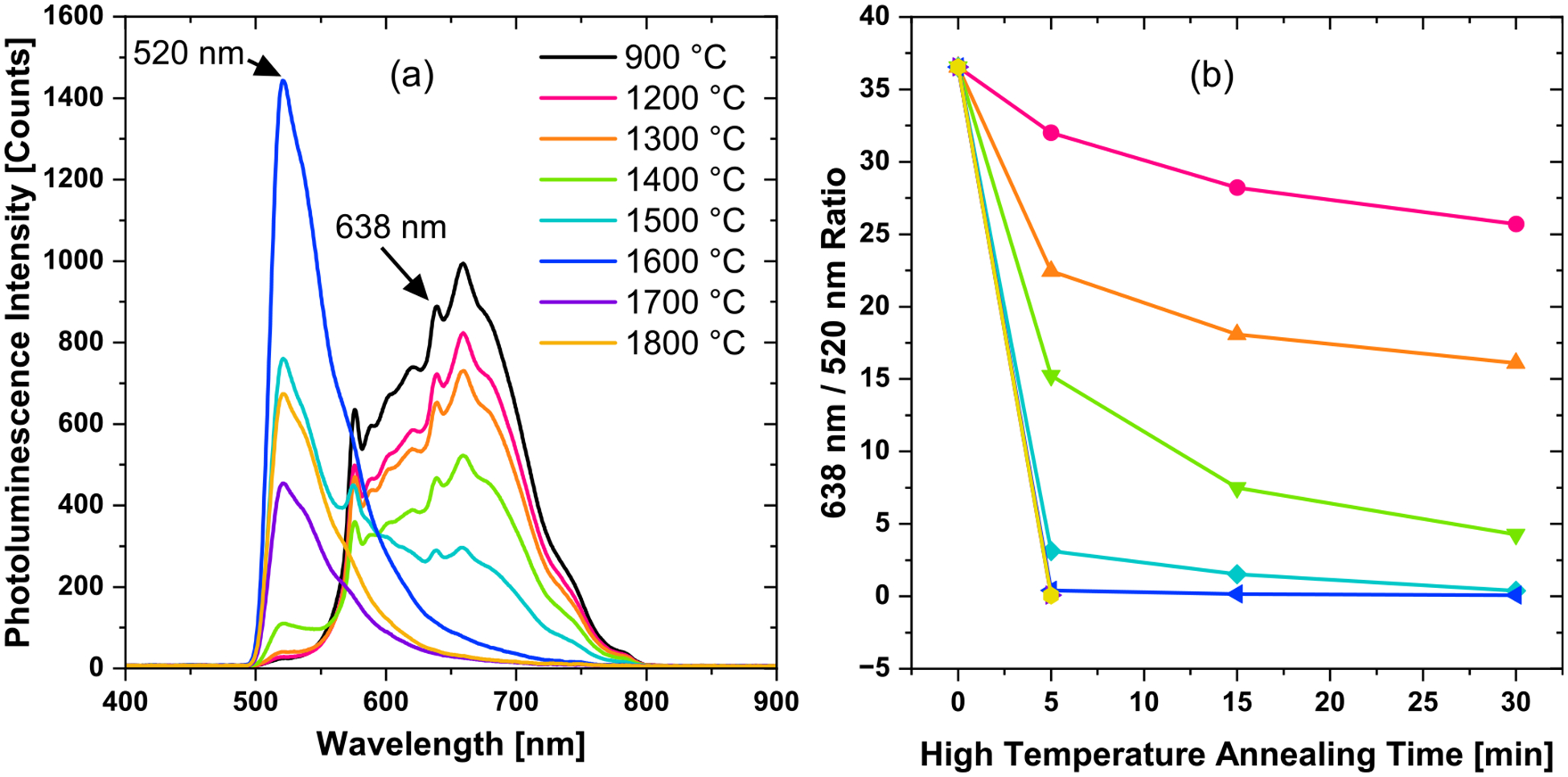
Photoluminescence spectra of 3 μm diamond particles under blue excitation after either 5 min (1700 °C and 1800 °C) or 30 min (series of temperatures from 1200 °C to 1600 °C) annealing **(a)**. Color coding of the temperatures is shown as an insert in **(a)**. **(b)** E Ratios of the PL intensities at 638 nm (NV^−^ zero phonon line) to the intensities at 520 nm (H3 and Ni Related Center) for all the annealing treatments and durations **(b)**. The ratio for the particles after e-beam irradiation and annealing at 900 °C for 2 h was used as a starting point (0 min high temperature annealing time). Lines are added as guides for the eye.

Changes in luminescent centers were monitored by PL spectroscopy. Two signals of interest are the peaks at 520 and 638 nm. These lines correspond to a sideband of the H3 (NVN) and/or Ni-related centers and the zero-phonon line (ZPL) of the NV^−^ center, respectively (Zaitsev, 2001). Figure 6a shows representative PL emission spectra of the diamond particles under blue light excitation for 30 min annealing treatments in the temperature range of 1400 °C–1800 °C as compared with the starting particles. This range of temperatures is where the most significant PL changes are observed. Figure 6b shows the ratio of the 638 nm ZPL corresponding to the NV- center to the 520 nm peak relative to the starting particles (900 °C–2 h). Increase in the annealing temperature results in a growth of the 520 nm line along with a suppression of NV^−^ line. This is indicative of a formation of H3 centers and/or Ni-related centers. A progressive decrease in the NV fluorescence emission with higher temperature annealing was observed. An example of a decrease in the fluorescence output for the 1600 °C annealing series is shown in Supplementary Figure S3 (Supplemental Information).

### Optically detected magnetic resonance and *T*_1_ relaxation

**FIGURE 7:**
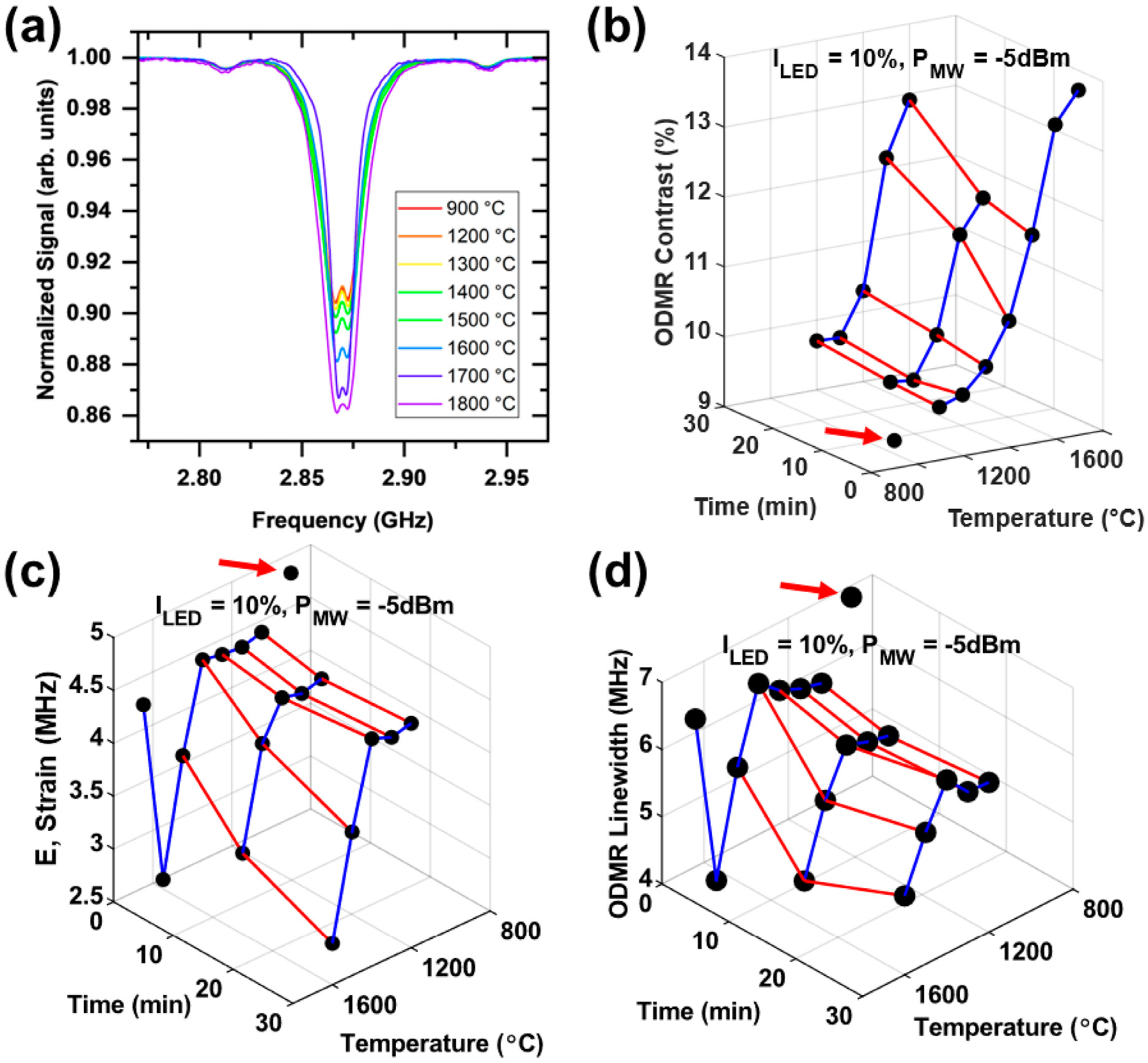
CW ODMR characterization of the annealed diamond particles. Representative ODMR spectra after 5 min annealing treatments **(a)**. ODMR spectral contrast **(b)**, strain (*E*-parameter) **(c)**, and full width at half maximum (FWHM) linewidth **(d)** as a function of treatment time and temperature. Black dots denoted with red arrows in **(b–d)** are the 900 °C/2 h annealed starting particles.

Figure 7 shows representative NV^−^ ODMR spectra (a) and three parameters derived from such spectra: (1) the contrast (b), (2) the strain splitting *E* (c), and (3) the linewidth (d). The following trends were observed: a progressive increase in the ODMR contrast starting with annealing temperatures of ≈1400 °C (Figure 7a,b); a decrease in the strain (*E*) at 1500 °C annealing and higher (Figure 7C); a decrease in the linewidth at the annealing temperatures of 1500 °C and above (Figure 7d). We note that the particles annealed at 1800 °C fall out of most of these trends. This is likely attributed to considerable structural damage/rearrangements of the diamond crystal structure resulting from an extensive graphitization at this temperature. Thus, high temperature annealing at temperatures as high as ≈1700 °C improves NV^−^ spin properties that manifest in enhancing ODMR contrast and reducing ODMR linewidth and the strain. The temperature onset of these improvements tends to coincide with the range where changes in EPR spectra and spin concentrations were also observed–generally, at 1500 °C and above (Table 1).

We speculate that the observed changes in ODMR spectra upon annealing at high temperatures are attributed to several factors. The first factor is a decrease in dipolar interaction between NV^−^ and other paramagnetic species present as the concentration of the latter decreases significantly upon annealing. This would result in an increased *T*_2_* and a reduced ODMR linewidth (Bauch et al., 2020). The second factor can be a reduced lattice strain, reflected by the strain parameter *E*, due to aggregation of nitrogen (Jones et al., 2015; Dobrinets et al., 2013). The NV^−^ center is an axially symmetric defect, and for such defects in the absence of external electric fields, the *E* parameter in ZFS spin Hamiltonian (Equation 9) should be zero, but off-axis strains in the lattice may distort the defect symmetry (Rondin et al., 2014).


(9) $$h^{-1}{\overset{ˆ}{ℋ}}_{\text{ZFS}}=D\left({\overset{ˆ}{S}}_{z}^{2}-\frac{1}{3}S(S+1)\right)+E\left({\overset{ˆ}{S}}_{x}^{2}-{\overset{ˆ}{S}}_{y}^{2}\right)$$

In Equation 9 *S* = 1 for the ground triplet state, *D* is the axial ZFS parameter (≈2.87 GHz), *E* is the off-axis ZFS strain parameter, *h* is the Planck constant, and ${\overset{ˆ}{S}}_{i}$ are projection spin operators. A reduction in *E* parameter suggests an improvement in the crystalline lattice quality.

**FIGURE 8:**
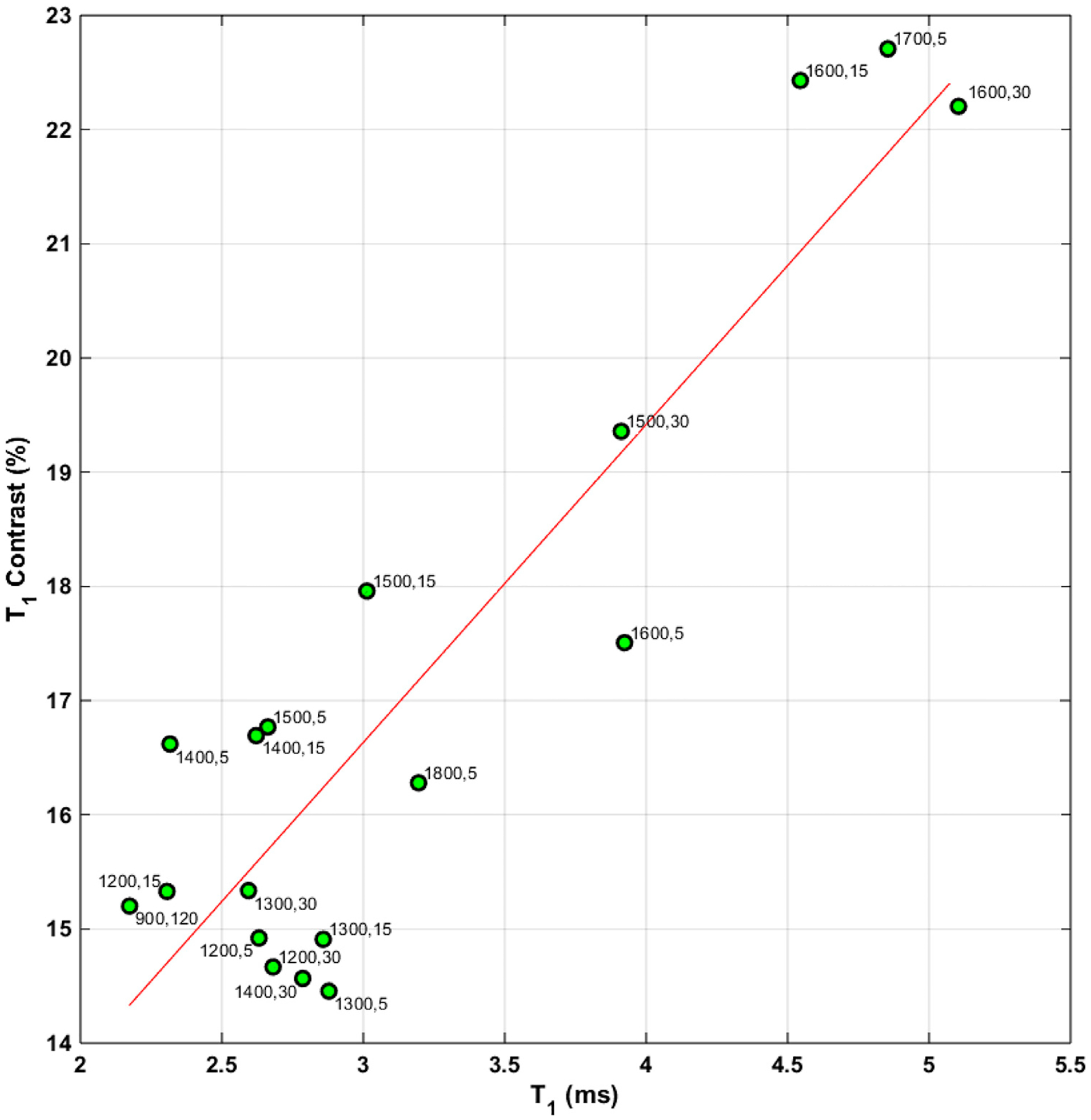
*T*_1_ contrast defined by Equation 2 as a function of *T*_1_ relaxation times for the various annealing temperatures.

ODMR relaxation measurements for NV^−^ centers showed *T*_1_ relaxation times within a narrow range from *ca*. 2.5–5 ms for all the samples, with longer *T*_1_ being observed for the samples annealed at higher temperatures. Annealing below *ca*. 1400 °C had a limited impact on the *T*_1_ times. Thus, as with CW ODMR data, *T*_1_ only seems to be affected when the annealing temperature and time are sufficient for nitrogen mobility. NV^−^ *T*_1_ relaxation at room temperature is predominantly caused by intrinsic processes such as phonon interactions and cross-relaxation effects between NVs and other spins (Jarmola et al., 2012). Thus, the observed increases in *T*_1_ are likely a result of a reduced concentration of NV and other spins after annealing at higher temperatures. Figure 8 examines *T*_1_ contrast (Equation 2) and *T*_1_ relaxation times for existence of a linear correlation. We note that the *T*_1_ contrast is an approximate measure of spin polarization, and, therefore, a positive correlation with the *T*_1_ times is expected. Again, the poor contrast of the 1800 °C annealed sample is probably a result of a significant graphitization, and we speculate that annealing treatments at this and higher temperatures are not increasingly beneficial for particles of this size.

### Impact of annealing on NV^−^thermometry noise level

Thermometry of NV^−^ defects in diamond is based on dependence of the triplet ground state ZFS parameter *D* ≈ 2.87 GHz on temperature. The ZFS decreases linearly with temperature with a proportionality constant $c_{T}=\frac{dD}{dT}\approx -74.2\;\text{kHz}/\text{K}$ at about room temperature (Acosta et al., 2010). One sensitive approach to measure changes in *D* is based on frequency-jump modulation between two discrete frequencies at the inflection points at both sides of the NV^−^ ODMR resonance line (Singam et al., 2020). The amplitude of the resulting modulated signal was shown to be proportional to the line temperature shift over a wide range of temperatures (Singam et al., 2020). For such a temperature sensing method, CW ODMR spectra from diamond particles exhibiting narrow lines and high contrast result in larger derivatives at inflection points of the central peak around *D* and, thus, improve sensitivity. Specifically, if an ODMR line is a Gaussian, then it follows immediately that the slopes at the inflection points are proportional to the ratio of the contrast to the linewidth.

**FIGURE 9:**
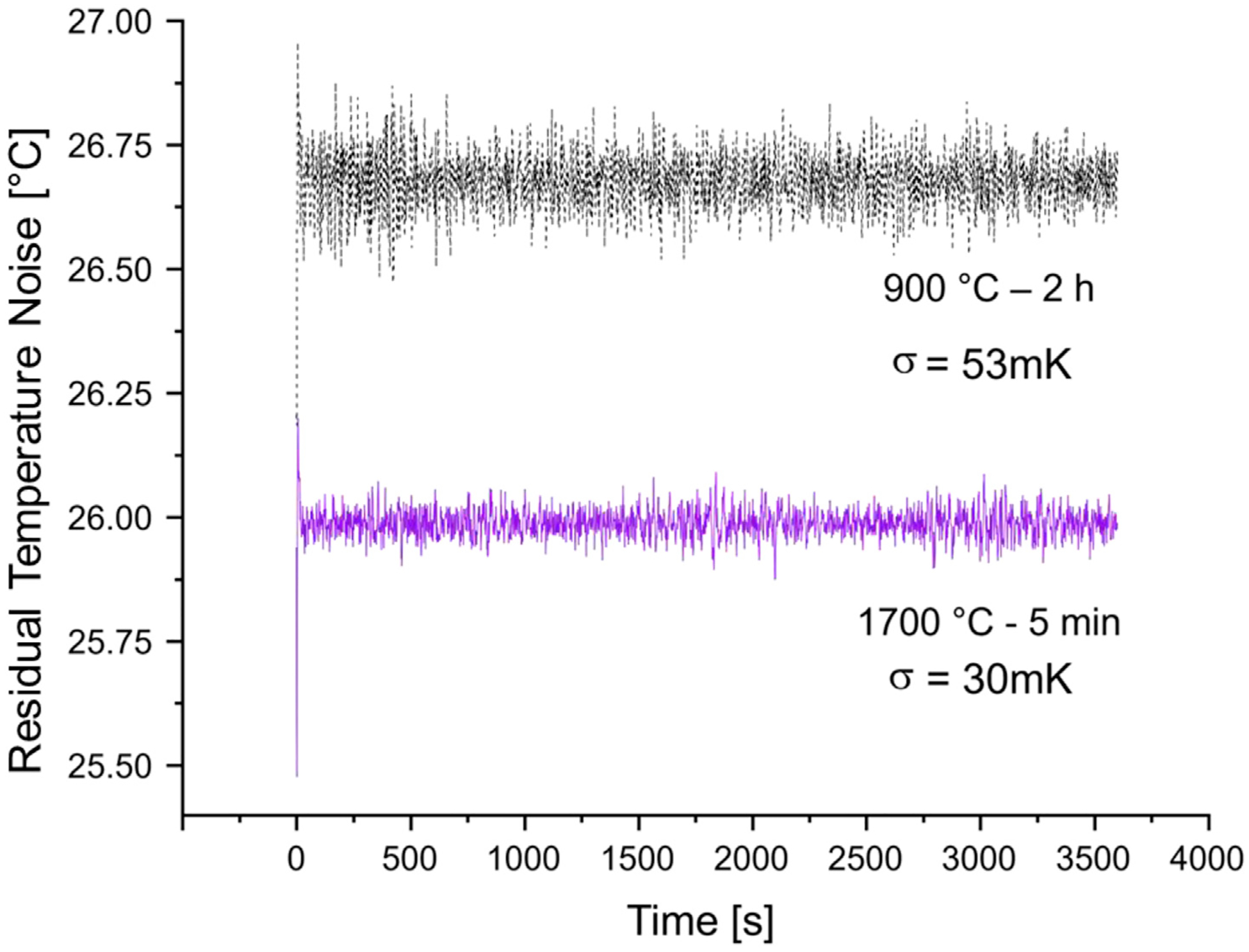
Residual temperature fluctuations of the NV^−^ thermometer after monitoring ambient temperature for 1 h with either the starting particles (900 °C–2 h) (black, dashed trace) or high temperature annealed particles (1700 °C–5 min) (purple, solid trace) after baseline subtraction of the raw signal (Supplementary Figure S4, Supplemental Information).

To demonstrate the benefits of high temperature annealing for NV^−^ thermometry using diamond particles, we have chosen to compare the particles annealed at 1700 °C for 5 min with the initial particles annealed for 2 h at 900 °C. The former particles exhibited the highest magnetic field contrast and the narrowest lines in the ODMR spectrum among all the samples characterized here. Figure 9 compares temperature recordings for the two samples and the noise floor for the particles at 1700 °C for 5 min is clearly lower. An improvement of temperature precision measurement, defined as $\left(\sigma_{900{}^{\circ}\text{C}}/\sigma_{1700{}^{\circ}\text{C}}-1\right)\times 100\%\approx 77\%$ was observed. For the 1700 °C - 5 min annealed particles the contrast was improved by *ca*. 32% while the linewidth decreased also by ≈ 24%. Then the slopes at the inflection pointes, as discussed earlier, increased by a factor of ((1 + 0.32)/(1 – 0.24)) ≈ 1.74 resulting in *ca*. 74% improvement in temperature precision. This predicted value closely aligns with 77% improvement observed experimentally.

## Conclusion

A comprehensive analysis of high temperature annealing treatments of 3 μm fluorescent diamond particles and the impacts of such treatments on NV^−^ spin properties was carried out. Annealing in the temperature range of *ca*. 1400 °C–1800 °C results in substantial changes to the concentrations of NV^−^ centers as well as P1 centers. Quantitative CW EPR data on concentration of NV^−^ and P1 centers for the series the annealed samples were analyzed to estimate activation energies for the annihilation of NV^−^ centers and the aggregation of P1 centers to form A-centers (N-N pairs). The activation energies of 3.89 eV and 3.63 eV for each process are remarkably similar. We speculate that this may be an indication of the same limiting step in the defect annihilation such as temperature-activated defect migration through the diamond lattice. The activation energies reported here fall in between literature values of vacancy diffusion (*ca*. 2.3 eV^41^) and vacancy-assisted nitrogen migration (*ca*., 4.5 eV^42^) although the latter values were measured at lower temperatures. NV^−^ spin properties were also assessed with CW ODMR and ODMR *T*_1_ relaxometry. Overall, NV^−^ spin properties improve by increasing the annealing temperatures from 1400 °C to 1700 °C. These improvements include a reduced ODMR linewidth, a decrease in the zero-field splitting strain parameter *E*, enhancement of ODMR signal and *T*_1_ contrast, and an increase in *T*_1_ from ≈2 ms to 5 ms–all measured at room temperature. Annealing at 1800 °C resulted in a degradation of these spin properties essential for quantum sensing, but this loss is most likely the result of an extensive graphitization induced at this temperature. A significant reduction of NV^−^ content after the annealing treatments at temperatures of 1500 °C and above, even for periods as short as 5 min, was also observed. This diminishes brightness substantially, meaning that the use of such particles for quantum sensing is better suited in a form of ensembles rather than single particles although laser-based excitation may mitigate this to some extent.

## Supplementary Material

Supplementary Information

The Supplementary Material for this article can be found online at: https://www.frontiersin.org/articles/10.3389/frqst.2025.1709220/full#supplementary-material

## Figures and Tables

**TABLE 1:** Particle yields and estimated primary, P1, and NV^−^ concentrations following different annealing protocols. Typical errors in spin concentrations do not exceed 15%.

Treatment	Yield (%)	Primary defect content (ppm)	P1 content (ppm)	NV^−^ content (ppm)
900 °C/2 h	97	58	51	1.1
1200 °C/5 min	86	57	50	1.0
1200 °C/15 min	77	58	52	1.1
1200 °C/30 min	86	58	51	1.1
1300 °C/5 min	79	58	51	1.1
1300 °C/15 min	83	58	52	1.1
1300 °C/30 min	60	57	51	1.0
1400 °C/5 min	61	57	51	1.0
1400 °C/15 min	n/a^a^	53	47	1.0
1400 °C/30 min	63	50	45	0.9
1500 °C/5 min	88	52	46	0.9
1500 °C/15 min	61	46	42	0.6
1500 °C/30 min	85	30	27	0.3
1600 °C/5 min	88	38	35	0.4
1600 °C/15 min	75	25	23	0.12
1600 °C/30 min	81	20	18	0.024
1700 °C/5 min	36	23	21	n/a^b^
1800 °C/5 min	1.6	n/a^c^	n/a^c^	n/a^c^

a This sample was partially lost during handling, so an accurate yield could not be determined.

b No NV^−^signal was detected by EPR.

c No EPR, spectra were measured because of insufficient amount of diamond particles recovered after the annealing.

## Data Availability

The raw data supporting the conclusions of this article will be made available by the authors, without undue reservation.
