# Supplementary material for: Optimizing ensemble NV− spin properties of fluorescent diamond microparticles by systematic low pressure high temperature annealing
Source: Front Quantum Sci Technol. Author manuscript; Available in PMC 2026 May 30. (PMC13220318; doi:10.3389/frqst.2025.1709220)
Supplement: Supplementary Information [file NIHMS2167572-supplement-Supplementary_Information.docx]

**Optimizing Ensemble NV^-^ Spin Properties of Fluorescent Diamond Microparticles by Systematic Low Pressure High Temperature Annealing**

Nicholas Nunn^1^, Antonin Marek^1,2^, Marco D. Torelli^1^, Alex I. Smirnov^2^, Olga A. Shenderova^1^

^1^Adámas Nanotechnologies, Inc., Raleigh, NC 27617, USA

^2^Department of Chemistry, North Carolina State University, Raleigh, NC 27695, USA

**Supporting Information**

**S1. Progressive Graphitization of Powder after High Temperature Annealing S3**

**S2. Estimated Area Power Density for the CoolLED Light Source S4**

**S3. Drop in NV Fluorescence Emission Intensity at 1600 °C S5**

**S4. Raw Temperature Monitoring Data S6**

**S1. High Temperature Annealed Particles after Vacuum Annealing**


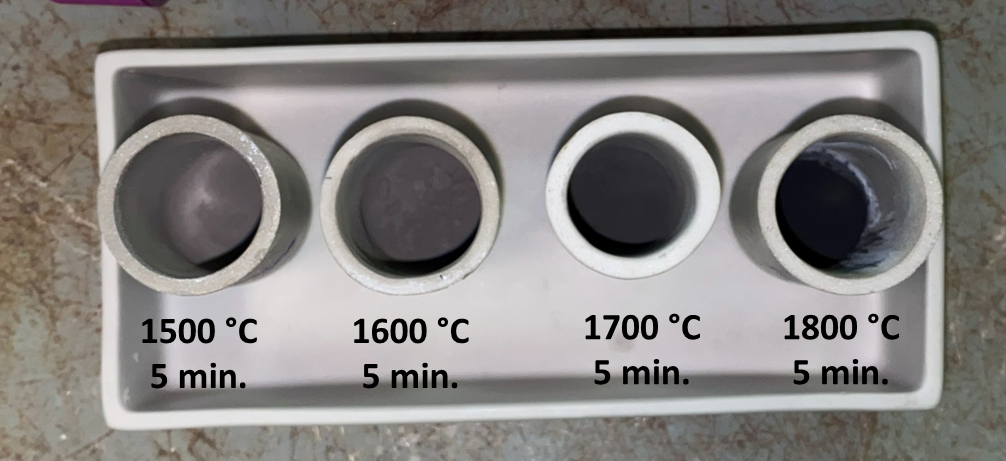


**Figure S1** A photograph of 3 µm diamond powder following high temperature annealing at 1500 ^°^C, 1600 ^°^C, 1700 ^°^C, and 1800 ^°^C for 5 min at each temperature. Progressive darkening of the powder indicates an increasingly higher graphitization as the annealing temperature increases.

**S2.** **Estimated Area Power Density for the CoolLED Light Source**


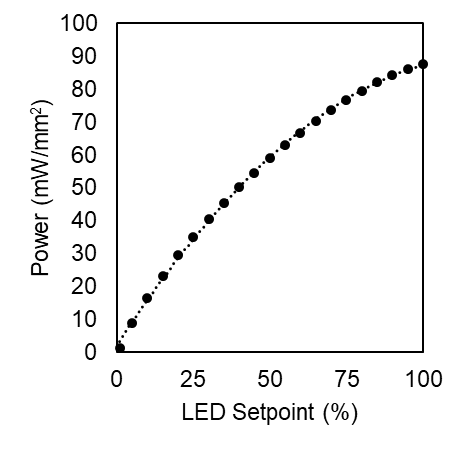


**Figure S2** Estimated areal power density versus LED setpoint for the pE300 CoolLED with a 40x objective having an estimated view area of 0.38 mm^2^.

**S3. Drop in NV Fluorescence Emission after 1600 °C Annealing**


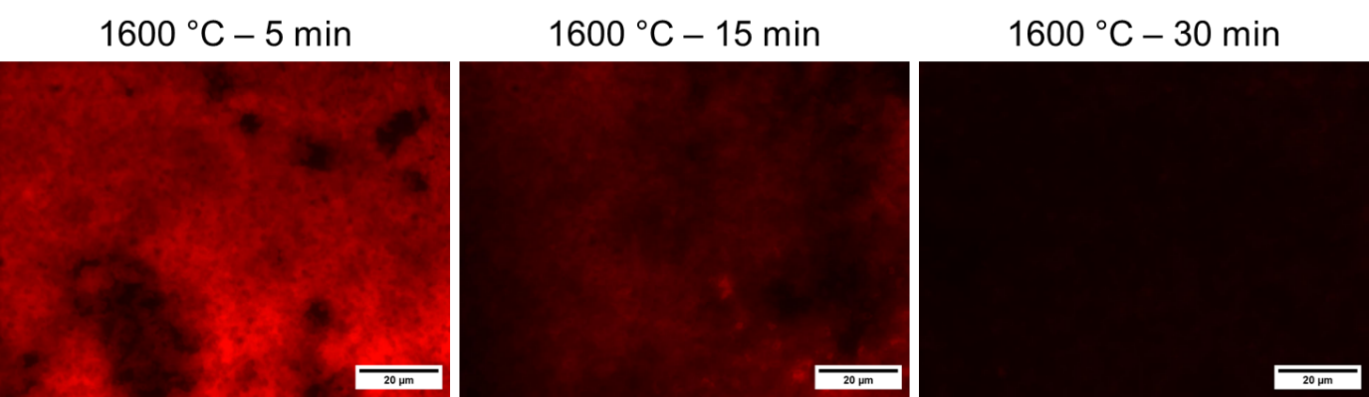


**Figure S3** Fluorescence images of an ensemble of1600 °C high temperature annealed 3 µm diamond particles under green excitation demonstrate a progressively reduced emission with the annealing duration. This observation is consistent with a progressively reduced NV content as measured by EPR. All images were captured at 64× magnification and 75 ms exposure time with a CCD camera (MT5000, AmScope).

**S4.** **Raw Temperature Monitoring Data**


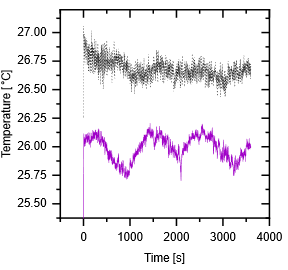


**Figure S4** Raw temperature data measured by NV^-^ thermometer before spline baseline subtraction show slow temperature changes in the lab. Ambient temperatures were monitored for 1 h using standard annealed particles (900 °C – 2 h, black dashed trace) or high temperature annealed particles (1700 °C – 5 min, purple solid trace).
